# Assessment of cyclists yielding to pedestrians at an unsignalized zebra crossing in Germany using drone video

**DOI:** 10.1038/s41598-025-20585-7

**Published:** 2025-11-04

**Authors:** Hiba Nassereddine

**Affiliations:** Faculty of Civil Engineering and Environmental Sciences, Institute of Transportation and Spatial Planning, Professorship for Traffic Psychology, University of the Bundeswehr Munich, Werner-Heisenberg-Weg 39, 85577 Neubiberg, Germany

**Keywords:** Cyclist-pedestrian interactions, Zebra crossing safety, Cyclist yielding behavior, Regression analysis, Clustering analysis, Drone video data, Civil engineering, Human behaviour

## Abstract

Previous research has examined vehicle-pedestrian and vehicle-cyclist interactions, but there have been few studies that examined cyclist-pedestrian interactions at intersections. This study addresses this gap by analyzing cyclist-pedestrian interactions at an unsignalized intersection in Germany using publicly available drone data. The study presents a framework and proof of concept for analyzing cyclist behavior proactively, without relying on crash data. The primary objectives are to identify the variables influencing cyclist yielding behavior and obstructed travel time (OTT) within a predefined zone at a zebra crossing and to classify cyclist behaviors. Using logistic and linear regression models, several key predictors were identified, including cyclist speed, trajectory changes, pedestrian time-to-conflict-point, and interaction proximity, which significantly impacted yielding behavior. Speed reduction and pedestrian presence on the zebra crossing were found to improve yielding rates. Additionally, clustering analysis revealed two optimal and distinct cyclist behavior groups: one cluster exhibiting less yielding behavior, while the other demonstrated greater compliance with traffic laws. This proactive approach provides a valuable alternative in environments where crash data acquisition is complicated by privacy regulations. It offers critical insights for traffic management strategies aimed at enhancing pedestrian safety at unsignalized intersections, making it applicable to broader contexts with similar data challenges.

## Introduction

Walking and cycling are popular modes for short-distance trips, promoting both sustainable transport systems and healthy lifestyles. Many national and local governments worldwide advocate for cycling to enhance sustainability^1–4^. Creating walkable and cyclable road networks aids in reducing private vehicle usage, addressing energy and environmental concerns^5^. However, the rise in urban cycling and walking has increased interactions between pedestrians and cyclists, which are critical to study due to their impact on safety, comfort, and urban transportation efficiency. Because the two groups move at comparable speeds, share sight lines, and have similar reaction times, conventional right-of-way cues become blurred, generating many near misses that do not appear in crash statistics. Understanding these interactions is therefore essential for increasing perceived pedestrian safety and for achieving Vision Zero goals that cover all road users, not only motorized traffic.

Cyclist-pedestrian interactions occur in various urban settings, including intersections, shared paths, and pedestrian crossings. These interactions range from simple co-presence to more complex scenarios involving yielding, stopping, or altering paths to avoid collisions. The nature of these interactions is influenced by infrastructure, road user behavior, and contextual elements such as traffic volume and environmental conditions. Traffic conflicts are defined as situations where two or more road users come so close in time and space that a collision is likely if neither altered course. Interactions broaden this definition to include less severe events, such as when a pedestrian and cyclist are on the road simultaneously and adjust their movements in advance to prevent a conflict, ensuring smooth movement for both^6^. Although these interactions increasingly affect urban comfort and safety, they have received less attention than vehicle-related interactions. Many reports document interactions between pedestrians and cyclists, but due to a lack of data, little is known about the frequency and nature of cyclist-pedestrian collisions, conflicts, and interactions. Collisions resulting in injury between pedestrians and cyclists are relatively rare^7–9^, making them difficult to study and often an insufficient data source for predicting new collisions.

Previous research has examined vehicle-pedestrian and vehicle-cyclist interactions, but there has been less studies on cyclist-pedestrian interactions^10^. The studies that have focused on cyclist-pedestrian interactions have identified various individual and environmental factors influencing these interactions, such as pedestrian gender, speed, distraction (e.g., cell phone use), pedestrian density, two-way roads, speed limits, and ground markings for cyclists^10–15^. Most of the existing research has concentrated on shared paths, with very little attention given to interactions at intersections. The limited studies that have examined interactions at intersections were conducted at non-signalized intersections on university campuses^11,14^, where behaviors may differ from those at various intersection types throughout a city. In support of this, the 2023 edition of the Cycling Monitor in Germany^16^ surveyed 4003 citizens aged 14 to 69 about their mobility preferences and habits. The survey revealed that 60% of respondents believe that many cyclists contribute to road traffic insecurity due to their driving behavior, with 53% identifying as cyclists. In the 2021 edition, 43% of respondents felt that other road users do not show enough respect and consideration for pedestrians. Among the 77% who identified as cyclists, 34% admitted to only following traffic rules they deemed appropriate.

Expectations that cyclists will yield to pedestrians at crossings are not always met, leading to potential safety issues. Cyclists often swerve or alter speed to avoid yielding, which can surprise pedestrians and lead to unsafe situations. In a Vancouver and Montreal study, cyclists yielded more consistently in well-designed environments with clear expectations and signals. Key factors influencing interactions included pedestrian gender, speed, and the presence of pedestrian ground markings^17^. Another study showed that 47.5% of interactions at pedestrian crossings occur between a conflicting pedestrian and a cyclist passing through, with both parties traveling perpendicular to each other. The study also reported that the most common issue concerning cyclists is the failure to give way to pedestrians at pedestrian crossings^18^.

Traditional methods for assessing intersection safety typically rely on historical crash data^19^. However, this approach has notable limitations, especially at unsignalized intersections where such data is often sparse, incomplete, or unavailable, necessitating alternative evaluation methods^20^. An alternative is the use of surrogate safety measures (SSMs), which indicate potential collision risks based on observable interactions rather than actual crashes^21^. Rather than relying on rare crashes, SSMs quantify the likelihood and severity of potential conflicts by analyzing observable interactions, such as relative timing, spacing, and evasive actions. SSMs use trajectories from video or sensors to enable proactive safety evaluations at sites where crash records are sparse or unrepresentative. SSMs also facilitate comparisons across locations and designs before harm occurs. Utilizing video analysis and advanced detection technologies, detailed data on pedestrian and cyclist movements can be captured to compute these SSMs^22^. Some studies have analyzed the time needed to complete a right turn based on a pedestrian’s position in the crosswalk to understand how drivers respond to pedestrians at signalized intersections. The time taken to complete a right turn was considered an effective safety indicator, reflecting the level of “respect” drivers have for pedestrians and serving as a surrogate safety measure to rank intersections by safety based on driver behavior, independent of crash data^23,24^. Another study of vehicle-pedestrian interactions at unsignalized intersections clustered driver behavior by using the obstructed travel time of through-moving vehicles in a predefined zone; it revealed two distinct groups, one with high driver-yielding compliance, and another with low compliance^25^. A pattern-based approach using SSMs has been proposed to enhance safety by educating drivers about proper behavior. This approach categorizes pedestrian-vehicle interactions based on road user behavior, particularly distinguishing between evasive and non-evasive behaviors of pedestrians and vehicles at unsignalized intersections^26^. These methods used to investigate vehicle-pedestrian interactions could also be applied to study cyclist-pedestrian interactions.

Cyclist-pedestrian interactions at unsignalized intersections remain understudied because reliable data are limited. Crash report capture only the rare events that end in injury or fatality, while systematic video recording is difficult, especially in Germany, where strict privacy rules and lengthy approval processes limit recording. Even when cameras are installed, footage suffers from occlusion, limited viewing angles, and the observer effect: road users modify their behavior when they notice conspicuous equipment. Consequently, although several studies have documented driver compliance at zebra crossings, reporting yielding rates that span from as low as 4% to as high as 45% in different national contexts^27–31^; by contrast, the yielding behavior of cyclists at comparable crossings has received far less scholarly attention.

To address these gaps, this study presents a framework and proof of concept for using drone video data from a top-down view, sourced from a publicly available dataset, as an alternative method for studying road user behavior proactively, without the need for crash data. The study leverages high-resolution drone footage shot from more than 60 m above the carriageway. The bird’s-eye perspective eliminates occlusions, delivers high positional accuracy in both longitudinal and lateral axes, and remains effectively invisible to people on the ground, preserving naturalistic behavior while masking personal identities. This approach provides a more dynamic understanding of cyclist-pedestrian interactions, facilitating safety improvements before crashes occur. By classifying and categorizing cyclist behavior during interactions with pedestrians at a zebra crossing within a predefined zone, this study aims to inform targeted interventions to improve safety and compliance with traffic laws. The specific objectives are:

1. to identify and understand the factors influencing cyclist yielding behavior and cyclist travel time when pedestrians are present, and.
2. to apply clustering techniques to categorize cyclists based on their interaction patterns, providing insights into different behaviors to pedestrians.

## Methodology

### Model-based clustering^32^

Model-based clustering is a statistical approach to partitioning data into clusters, where each cluster is assumed to be generated by a particular probabilistic model. This methodology offers a principled framework for clustering by formally defining clusters through statistical models, often Gaussian mixtures.

In model-based clustering, the data is assumed to come from a mixture of underlying probability distributions, where each component of the mixture corresponds to a cluster. The clustering problem is therefore transformed into a density estimation problem. Mathematically, if we denote the data by $\:X=\left\{{x}_{1},{x}_{2},\dots\:,{x}_{n}\right\}$, the density of $\:{x}_{i}$ is modeled as in Eq. (1).

1 $$\:f\left({x}_{i}|\varTheta\:\right)=\sum\limits_{k=1}^{K}{\pi\:}_{k}{f}_{k}\left({x}_{i}\right|{\theta\:}_{k})$$

where:

- $\:k$ is the number of clusters,
- $\:{\pi\:}_{k}$ is the mixing proportion for cluster k (with $\:{\sum\:}_{k=1}^{K}{\pi\:}_{k}=1\:$and $\:{\pi\:}_{k}>0$ for all $\:k$),
- $\:{f}_{k}\left({x}_{i}\right|{\theta\:}_{k})$ is the component density of the k^th^ component,
- $\:\varTheta\:={\left\{{\pi\:}_{k},{\theta\:}_{k}\right\}}_{k=1}^{K}$ represents the parameters of the mixture model.

For example, in a dataset with two clusters, each cluster might be represented by a different Gaussian distribution, where $\:{f}_{k}\left({x}_{i}\right|{\theta\:}_{k})$ could be the probability density function of a Gaussian distribution with parameters $\:{\theta\:}_{k}$ (mean and covariance). The goal is to estimate these parameters $\:\varTheta\:$, which involves determining the mixing proportions $\:{\pi\:}_{k}$​ and the parameters of the component densities $\:{\theta\:}_{k}$​.

The data point $\:{x}_{i}$​ represents an individual observation, that has data on $\:d$ variables, in the dataset $\:X$, which is assumed to be generated from one of the $\:K$ clusters according to the probabilities $\:{\pi\:}_{k}$​. The challenge is to infer which cluster each data point belongs to and to estimate the parameters that best describe the data’s underlying distribution.

### Model-based clustering for mixed data^33^

Model-based clustering for mixed data extends these principles to datasets containing both continuous and categorical variables. For mixed data, each observation $\:{x}_{i}=({x}_{ic},\:{x}_{id})$ consists of continuous variables $\:{x}_{ic},\:$​ and discrete variables $\:{x}_{id}$​. The mixture model will include different types of distributions for continuous and categorical variables. One approach is to use a latent class model for categorical data and a Gaussian model for continuous data, with joint density specified as in Eq. (2):

2 $$\:f\left({x}_{i}|\varTheta\:\right)=\sum\limits_{k=1}^{K}{\pi\:}_{k}{f}_{kc}\left({x}_{ic}\right|{\theta\:}_{kc}\left){f}_{kd}\right({x}_{id}\left|{\theta\:}_{kd}\right)$$

where:

- $\:{f}_{kc}$ is the probability density function (pdf) of the continuous variables for cluster k,
- $\:{f}_{kd}$​ is the probability mass function (pmf) of the discrete variables for cluster k,
- $\:{\theta\:}_{k}=({\theta\:}_{kc},\:{\theta\:}_{kd})$ are the parameters for the continuous and discrete parts of cluster k.

### Estimation and model selection

The Expectation-Maximization (EM) algorithm is a widely used method for estimating the parameters of mixture models. During the E-step, the algorithm calculates the expected value of the log-likelihood based on the current estimates of the distribution over cluster assignments. In the M-step, it maximizes this expected log-likelihood to update the parameter estimates.

In practice, the EM algorithm starts with initial parameter guesses and alternates between the E-step and M-step. This iterative process continues until the parameters converge to stable values, indicating that the mixture model has been adequately fitted to the data. Due to its flexibility, the EM algorithm is applicable to a broad range of clustering problems, from simple Gaussian mixtures to more complex scenarios involving different data types and distributions^32^.

The combination of EM for parameter estimation and criteria like the Bayesian Information Criterion (BIC) for model selection makes this methodology robust and efficient for uncovering the underlying structure in data. This ensures that the most appropriate model is chosen for representing the given dataset.

### Linear regression^34^

Linear regression is a fundamental statistical method used to model the relationship between a dependent variable and one or more independent variables. The goal is to find a linear equation that best predicts the dependent variable based on the independent variables. The simplest form is simple linear regression, where there is one independent variable. The model is expressed as in Eq. (3).

3 $$\:Y={\beta\:}_{0}+{\beta\:}_{1}X+\epsilon$$

where:

- $\:Y$ is the dependent variable,
- $\:X$ is the independent variable,
- $\:{\beta\:}_{0}$​ is the y-intercept,
- $\:{\beta\:}_{1}$ is the slope of the line, and.
- $\:\epsilon$ represents the error term, accounting for the variation in $\:Y$ not explained by $\:X$.

The coefficients $\:{\beta\:}_{0}$​ ​and $\:{\beta\:}_{1}$ are estimated using the method of least squares, which minimizes the sum of the squared differences between the observed values and the values predicted by the model. The least squares estimates are given by Eqs. (4) and (5).

4 $$\:\widehat{{\beta\:}_{1}}=\frac{{\sum\:}_{i=1}^{n}\left({X}_{i}-\bar{X}\right)\left({Y}_{i}-\bar{Y}\right)}{{\sum\:}_{i=1}^{n}{\left({X}_{i}-\bar{X}\right)}^{2}}$$

5 $$\:\widehat{{\beta\:}_{0}}=\bar{Y}-\widehat{{\beta\:}_{1}}\bar{X}$$

where $\:\bar{X}$ and $\:\bar{Y}$ are the means of $\:X$ and $\:Y$, respectively.

Multiple linear regression extends this to multiple independent variables. The model is given by Eq. (6).

6 $$\:Y={\beta\:}_{0}+{\beta\:}_{1}{X}_{1}+{\beta\:}_{2}{X}_{2}+\cdots\:+{\beta\:}_{p}{X}_{p}+ϵ$$

Here, $\:{\beta\:}_{j}$​ (for $\:j=1,\dots\:,p$) are the coefficients for the independent variables $\:{X}_{j}$.

### Logistic regression^35^

Logistic regression is used for modeling binary outcome variables, where the dependent variable can take on only two possible outcomes (often coded as 0 and 1). Unlike linear regression, logistic regression models the probability that $\:Y$ belongs to a particular category. The logistic regression model is formulated as in Eq. (7).

7 $$\:logit\left(P\left(Y=1|X\right)\right)=ln\left(\frac{P\left(Y=1|X\right)}{1-P\left(Y=1|X\right)}\right)={\beta\:}_{0}+{\beta\:}_{1}{X}_{1}+{\beta\:}_{2}{X}_{2}+\dots\:+{\beta\:}_{p}{X}_{p}$$

The left-hand side, $\:logit\left(P\left(Y=1|X\right)\right)$, is the log-odds of the probability that $\:Y=1$. The model ensures that the predicted probabilities lie between 0 and 1. The probability $\:P\left(Y=1|X\right)$ can be derived by applying the logistic function given by Eq. (8).

8 $$\:P\left(Y=1|X\right)=\frac{1}{1+{e}^{-\left({\beta\:}_{0}+{\beta\:}_{1}{X}_{1}+{\beta\:}_{2}{X}_{2}+\dots\:+{\beta\:}_{p}{X}_{p}\right)}}$$

The coefficients $\:{\beta\:}_{0},\:{\beta\:}_{1},{\beta\:}_{2},\dots\:,{\beta\:}_{p}$​ are typically estimated using maximum likelihood estimation (MLE), which finds the parameter values that maximize the likelihood function given by Eq. (9).

9 $$\:L\left(\beta\:\right)=\prod\limits_{i=1}^{n}{P\left({Y}_{i}|{X}_{i}\right)}^{{Y}_{i}}{\left[1-P\left({Y}_{i}|{X}_{i}\right)\right]}^{1-{Y}_{i}}$$

### Incorporating regression in clustering^36^

To enhance the analysis, regression models can be incorporated within the clusters identified by a clustering algorithm. Initially, the data is clustered based solely on specific key variables using a clustering algorithm, such as Mclust^37^. This initial clustering identifies distinct clusters within the dataset. Following this, separate regression models are fitted within each cluster to understand the relationship between multiple predictors and the response variable. The predictors used in the multiple linear regression include significant predictors identified from prior analysis.

This advanced approach integrates a regression model within each cluster, fitting a unique regression model for each cluster to predict the response variable based on the predictor variables. The combined methodology provides deeper insights and more precise data partitioning, allowing for the identification of distinct subgroups within the data, each characterized by its specific relationship between the response and predictor variables.

By leveraging the combined strengths of clustering and regression, this methodology offers a robust framework for analyzing complex datasets with mixed data types and inherent relationships between variables. It improves the interpretability of the clusters by providing clear mathematical relationships within each cluster, facilitating better decision-making and predictive analytics. This approach enhances the analytical power and applicability of clustering techniques, making it a valuable tool for researchers and practitioners alike.

### Data collection

Germany’s strict data-privacy laws mean that any video recording containing identifiable information, such as faces or license plates, requires explicit permission from local authorities. This process not only takes significant time but also requires full compliance with various data protection guidelines under the General Data Protection Regulation (GDPR). Given these challenges and the time-consuming nature of obtaining these permissions, for the purpose of this paper as a framework and proof of concept, the study relies on a publicly available dataset based on top-view drone video footage. The top-view perspective ensures that individuals are not directly identifiable, thus minimizing privacy concerns while still providing valuable data for analyzing cyclist-pedestrian interactions.

**Fig. 1:**
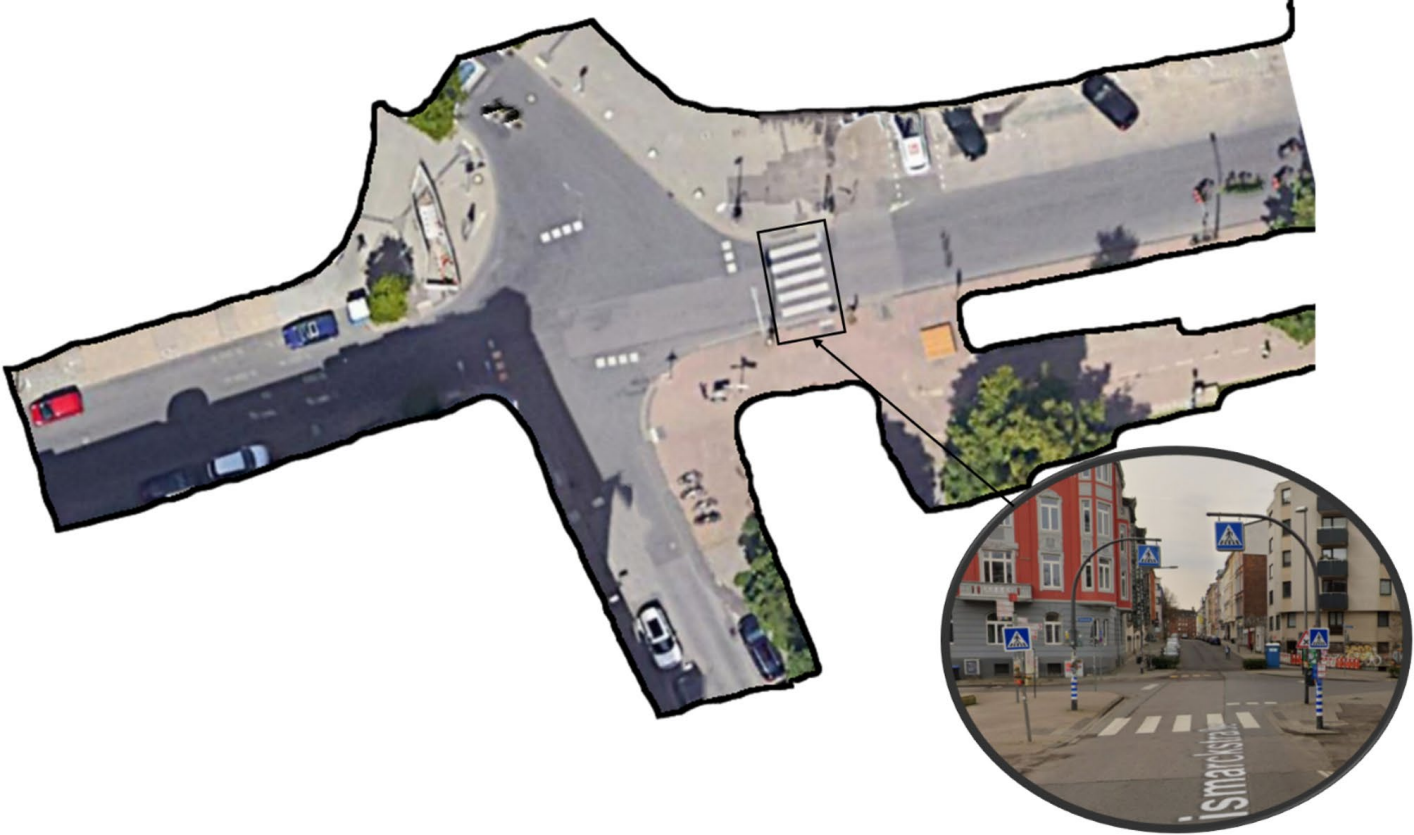
Google Maps satellite view of the unsignalized intersection of Bismarck Str. and Schloss Str. with visual representation of road users and zebra crossing signage (Imagery ©2025 Airbus, Map data ©2025 GeoBasis-DE/BKG (©2009), Google).

This study leverages a publicly available dataset^38^ to analyze and cluster cyclist behavior at an unsignalized intersection in Germany. The drone videos, captured in 4 K resolution (4096 × 2160 pixels) at 25 frames per second, each last about 20 min and cover an area of 80 × 40 m. A total of 242.6 min were recorded. Pedestrian and cyclist trajectories were provided by the dataset, which extracted and processed the trajectories from the drone video recordings at four intersections. This study focused on the intersection of Bismarck Str. and Schloss Str. (Recording ID 18 to 29), specifically examining the interactions between cyclists and pedestrians at a zebra crossing as shown in Fig. 1. The crossing at the intersection is 5 m long and 3.5 m wide, with zebra stripes marking the pedestrian crossing area. Pedestrian crossing signs are placed on both sides of the road, and a dotted line is present after the crossing to indicate a yield for vehicles entering the intersection. However, this yield line is intended for vehicles, not specifically for pedestrian right-of-way. The speed limit at this intersection was 50 km/h (30 mph).

While the raw video data was not provided, a Python source code was made available by the dataset provider to visualize the trajectories. The visualizer imports the trajectory data and displays it on an image of the recording site. Users can visualize specific frames or playback the recorded tracks, with the option to display information such as track IDs. Each road user class is represented by a specific shape: vehicles are displayed as rectangles, while cyclists and pedestrians are represented by dots for each frame they are visible.

Each recording consists of an image from the drone’s perspective and three CSV files. Two CSV files contain metadata about road users and the recordings, including details such as recording number, track ID, class, frame rate, duration, location, and the number of pedestrians. The third CSV file provides trajectory information per frame, featuring variables such as ID, frame number, x and y positions, x and y velocities, heading, x and y accelerations, and lateral and longitudinal velocities and accelerations. The dataset covers the trajectories of various vehicles (cars, buses, trucks) and vulnerable road users (pedestrians and cyclists), with the focus of this study being on pedestrian and cyclist trajectories. The age and gender of the road users were not included in the provided dataset.

The final dataset used for modeling cyclist-pedestrian interactions includes observations limited to leading cyclists passing through, i.e., the first rider in any approach stream, who encountered a single pedestrian or a pedestrian group crossing from one direction. Trailing cyclists, who might react to both the actions of the leading cyclist and the pedestrians, were excluded from the analysis. Likewise, if a motor-vehicle was present either directly ahead of or behind the cyclist, or pedestrians were crossing from both directions, these interactions were dropped so that the analysis isolates pure cyclist-pedestrian interactions. By excluding influences such as traffic volume, pedestrian density, and cyclist-cyclist interactions, this filtering approach confines the study to comparable one-to-one cyclist-pedestrian interactions allowing for a more focused analysis of the cyclist-pedestrian interaction at the crossing.

Interactions between cyclists passing through and conflicting pedestrians were documented within a predefined zone. The cyclist travel zone measures 30 m (approximately 100 ft) in length, starting 5 m (16.5 ft) before the pedestrian crossing and extending 25 m beyond it. The pedestrian crossing region was defined to include both the zebra crossing and areas outside of the crossing. A custom Python code was written to extract specific details, including the pedestrian’s start and end of crossing, the cyclist’s entry and exit from the predefined zone, and the direction of travel for both pedestrians and cyclists. Several other variables were also extracted and documented, including the number of conflicting pedestrians and whether the pedestrian(s) started crossing within the zebra crossing, among others.

An automated process was employed to identify interactions between cyclists and pedestrians. The automated Python script first flagged every potential cyclist-pedestrian interaction. Each candidate interaction was then visually checked frame-by-frame to confirm that one leading cyclist interacted with a single stream of pedestrians. Once an interaction was confirmed, the exact frames where the interaction occurred were analyzed.

To limit subjectivity, interaction confirmation followed two binary inclusion rules: (i) a single leading cyclist interacting with one pedestrian or a single pedestrian stream within the predefined zone, and (ii) no observable motor-vehicle influence on the cyclist-pedestrian interaction (e.g., no vehicle directly ahead/behind the cyclist within the zone or encroaching on the crossing). All machine-flagged candidates were reviewed frame-by-frame using the trajectory visualizer. Ambiguous cases, such as trailing cyclists, overtaking or side-by-side cyclists, simultaneous pedestrian streams approaching from both sides, partial occlusions around key timestamps, or any potential motor-vehicle influence, were excluded rather than resolved by judgment. No inferences about intent were made; decisions were based solely on observable criteria (leading status, single pedestrian stream, and absence of vehicle influence). A simple screening log was maintained to document decisions (candidate ID, keep or drop, and reason). To enhance reproducibility, future studies should include blinded duplicate screening of a sample of cases and report an inter-rater agreement statistic.

On two mid-weekdays in July 2019 (Tuesday and Wednesday), drones operated from 16:00 to 19:15 on day 1 and 16:00 to 18:00 on day 2; however, only 135 min and 104 min of footage, respectively, were recorded and provided for analysis. The weather was sunny/clear, and sunset has not yet occurred, so visibility and pavement conditions were stable. A total of 604 pedestrians crossing on the zebra crossing and 1,125 cyclists passing through were recorded during the 242.6 min of video data. Of these, 107 cyclist-pedestrian interactions of interest were identified. Table 1 summarizes the key characteristics of the video recordings used in the analysis, including the number of crossing pedestrians, passing cyclists, and identified interactions of interest during each recording session. Although the data was collected on different weekdays and times, the strict inclusion rules described above, together with the absence of darkness or adverse weather conditions, minimize the influence of time of day on the behavioral variables analyzed here. Residual temporal effects are acknowledged as a limitation and motivation for future multi-period data collection.

**Table 1:** Summary of video recordings and interactions of interest.

Day	Duration (min)	Number of crossing pedestrians	Number of passing through cyclists	Number of interactions of interest
Day 1	135.16	335	620	54
Day 2	104.40	269	505	53

Four cases of cyclist-pedestrian interactions were identified at the zebra crossing. These cases were defined by the direction of travel for both the cyclist and the pedestrian. The identified cases are:

- **case 1**: Cyclist traveling in direction A and pedestrian crossing in direction A,
- **case 2**: Cyclist traveling in direction A and pedestrian crossing in direction B,
- **case 3**: Cyclist traveling in direction B and pedestrian crossing in direction A, and
- **case 4**: Cyclist traveling in direction B and pedestrian crossing in direction B.

**Fig. 2:**
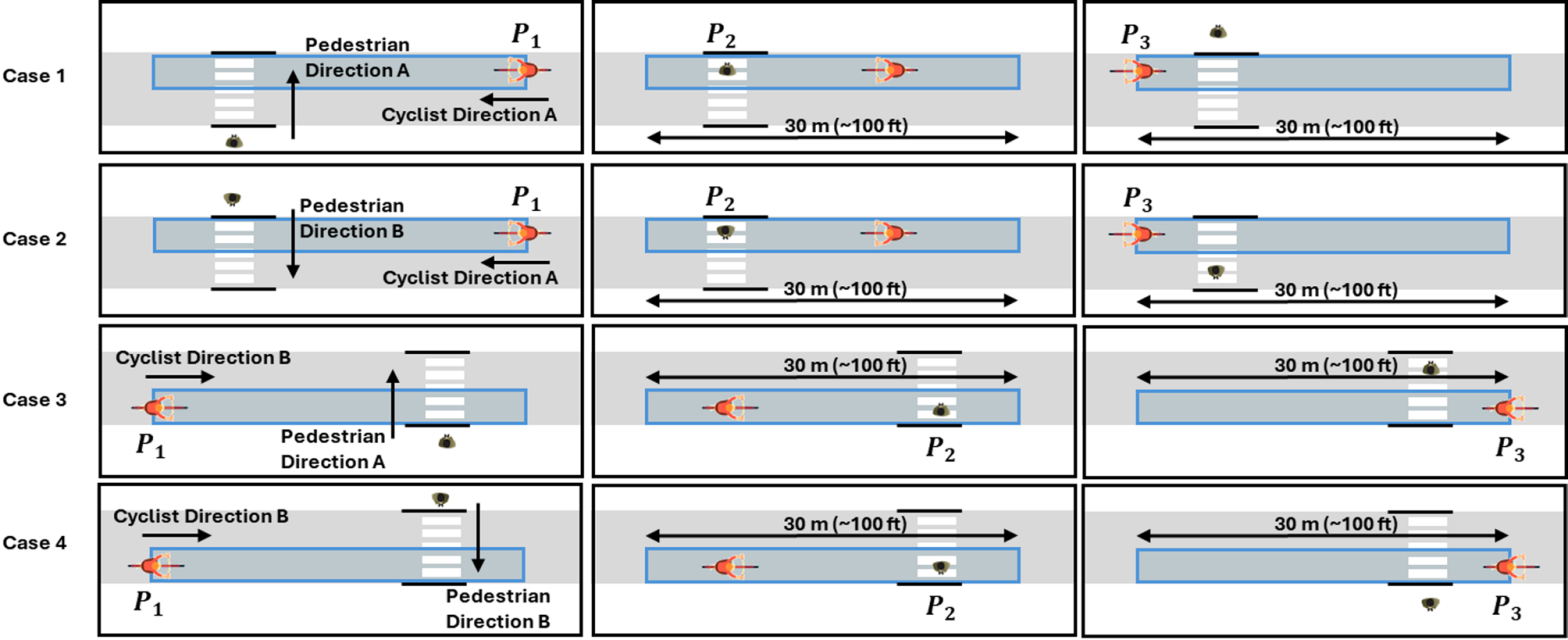
Visual representation of cyclist-pedestrian interaction cases and key timestamps.

The frame numbers from the dataset were converted into timestamps to capture the key moments of the interactions. Figure 2 illustrates these four identified cases, showing the cyclist travel zone along with the timestamps corresponding to the two key positions of cyclists and one key position of conflicting pedestrians:

- the timestamp when the center of the bicycle entered the predefined zone, referred to as $\:{P}_{1}$,
- the timestamp when a conflicting pedestrian arrived at the conflict point, referred to as $\:{P}_{2}$, and
- the timestamp when the center of the bicycle exited the predefined zone, referred to as $\:{P}_{3}$.

The four cases shown in Fig. 2 simply illustrate every geometric configuration that can occur when a single leading cyclist and a single pedestrian (or a pedestrian group) approach the zebra crossing from either side. Cases 1 and 4, as well as Cases 2 and 2, are mirror images that differ only in approach direction, but they are illustrated separately to show where the three reference timestamps $\:{P}_{1}$, $\:{P}_{2}$, and $\:{P}_{3}$ are located. In the analysis, direction is captured by a single binary variable (A or B).

### Data analysis and results

The Pedestrian Time to Conflict Point (TTCP) is defined as the time from when the cyclist enters the zone to when the pedestrian arrives at the conflict point ($\:TTCP={P}_{2}-{P}_{1}$), conceptually related to time-to-zebra (TTZ). The cyclist Obstructed Travel Time (OTT) is defined as the time it takes for a cyclist to traverse the zone ($\:OTT={P}_{3}-{P}_{1}$), adapted from Obstructed Right-Turn Time (ORTT)^23,24^. TTCP captures the temporal opportunity for interaction, and OTT reflects the cyclist’s behavior within that opportunity. These SSMs are used to indicate interaction dynamics and yield rather than to predict crash risk. To understand cyclist-pedestrian interactions, both logistic and linear regression models were applied to identify the key predictors of yielding behavior and obstructed travel time (OTT). The predictors considered in these models included pedestrian time-to-conflict-point (TTCP), cyclist speed upon entering the zone, cyclist deceleration, cyclist direction of travel, changes in path, speed reduction, the proximity of the interaction, whether the pedestrian was already on the zebra crossing, pedestrian speed, pedestrian direction of travel, whether the pedestrian started crossing within the zebra crossing, and whether the pedestrian was part of a group. A significance level of α = 0.05 was used to determine the inclusion of variables in the models.

Additionally, two clustering methods—Mclust and ClustMD—were applied to the dataset. These methods were chosen to capture different behavioral patterns due to the mixed nature of the data (continuous and categorical variables). Mclust assumes data follows a Gaussian distribution, while ClustMD incorporates a mixture of continuous and categorical variables. This approach ensured robustness in the clustering analysis, revealing distinct groups of cyclist behaviors based on interactions with pedestrians.

### Data summary

The time difference between when cyclists entered and exited the predefined travel zone ($\:{P}_{3}-{P}_{1}$) was calculated and referred to as the cyclist’s obstructed travel time (OTT), representing the duration a cyclist was impeded by a pedestrian. Similarly, the time difference between $\:{P}_{2}$ and $\:{P}_{1}$ was calculated as the pedestrian time-to-conflict-point (TTCP), which indicates how long it would take for a conflicting pedestrian to reach the conflict point when the cyclist was at $\:{P}_{1}$. In addition to OTT and TTCP, several other key variables were documented for each observation, including:

- the speed of cyclists entering the predefined zone ($\:{V}_{cyclis{t}_{entering}}$),
- cyclist deceleration ($$\:{Dec}_{cyclist}$$),
- pedestrian speed ($$\:{V}_{ped}$$),
- cyclist direction of travel ($\:{Dir}_{cyclist}$),
- pedestrian direction of travel ($\:{Dir}_{ped}$),
- whether the pedestrian started crossing within the zebra crossing ($\:{Cross}_{inside}$),
- whether a group of two or more pedestrians crossed together ($\:{Group}_{ped}$),
- whether the pedestrian was on the zebra crossing when the cyclist was 5 m (16.4 ft) away from the crossing ($\:PedOnZebra$),
- whether the cyclist changed their trajectory to avoid colliding with the conflicting pedestrian, that is a cyclist executed a swerve, either remaining on a curved path or returning to a straight line after the maneuver ($\:TrajectoryChang{e}_{cyclist}$),
- whether the cyclist reduced their speed when they were 5 m (16.4 ft) away from the crossing ($\:{SpeedReduction}_{cyclist}$), and.
- the proximity of the interaction, classified as “close” (both road users were near the conflict point) or “far” (one user was distant from the conflict point) ($\:{Interaction}_{Proximity}$).

Tables 2 and 3 provides a summary of selected statistics for these variables. This comprehensive dataset serves as the basis for the regression analyses, offering valuable insights into cyclist and pedestrian behavior at the zebra crossing, which can inform future traffic management strategies and safety improvements.

**Table 2:** Summary of continuous variables used in cyclist-pedestrian interaction modeling.

Variables	Min	Max	Mean
$\:OTT\:\left(sec\right)$	3.28	16.64	8.48
$\:TTCP\:\left(sec\right)$	2.28	12.60	6.74
$\:{V}_{{cyclist}_{entering}}\:\left(m/s\right)$	2.21	9.00	5.20
$\:{Dec}_{cyclist}\left(m/{s}^{2}\right)$	–2.85	–0.02	–0.96
$\:{V}_{ped}\left(m/s\right)$	0.42	3.30	1.57
Number of observations	107		

**Table 3:** Summary of categorical variables used in cyclist-pedestrian interaction modeling.

Variable	Category	Count	Percentage
$\:{Dir}_{cyclist}$	A	49	46%
	B	58	54%
$\:{Dir}_{ped}$	A	46	43%
	B	61	57%
$\:{Cross}_{inside}$	Yes	93	87%
	No	14	13%
$\:{Group}_{ped}$	Yes	27	25%
	No	80	75%
$\:PedOnZebra$	Yes	63	59%
	No	1	16%
	Ped started crossing	27	25%
$\:{TrajectoryChange}_{cyclist}$	Yes	23	21%
	No	84	79%
$\:{SpeedReduction}_{cyclist}$	Yes	67	63%
	No	40	37%
$\:Interactio{n}_{Proximity}$	Close	81	76%
	Far	26	24%
$\:{Yield}_{cyclist}$	Yes	63	59%
	No	44	41%

The dataset comprises 107 observations of cyclist-pedestrian interactions at a zebra crossing. Key metrics include the cyclist obstructed travel time (OTT), which averages 8.48 s, and the pedestrian time-to-conflict-point (TTCP), with a mean of 6.74 s. These metrics indicate the typical duration cyclists are impeded by pedestrians and the time pedestrians take to reach the conflict point, respectively. Cyclists entered the predefined zone at an average speed of 5.20 m/s and decelerated by an average of –0.96 m/s² when interacting with pedestrians. Pedestrian speeds varied, averaging 1.57 m/s, reflecting a range of pedestrian behavior. A notable finding is that 87% of pedestrians started crossing within the marked zone, and 25% were in groups, highlighting common pedestrian behaviors at zebra crossings. Additionally, 63% of cyclists reduced their speed, and 21% altered their trajectory during interactions, suggesting significant caution exercised by cyclists in the presence of pedestrians. However, 41% of cyclists did not yield to pedestrians, indicating a substantial portion of non-compliance with yielding behavior at zebra crossings. This lack of yielding behavior represents a significant safety concern and highlights the need for improved traffic management strategies and enhanced safety measures at unsignalized intersections. These insights underscore the importance of targeted interventions to increase cyclist compliance and protect pedestrian safety.

The video observations of the intersection revealed that among the cyclists who yielded to pedestrians, 29% did so when the pedestrians were still approaching the zebra crossing and had not yet started crossing. Furthermore, among the cyclists who yielded to pedestrians, 14% changed their trajectory path right before yielding. Among the cyclists who did not yield to pedestrians, 45% had close interactions with pedestrians. Additionally, among the cyclists who did not yield to pedestrians, 75% did not yield when the pedestrians were already on the zebra crossing.

### Logistic regression analysis

The logistic regression analysis aimed to model the probability of cyclists yielding to pedestrians at a zebra crossing. Initially, all potential variables influencing a cyclist’s decision to yield to pedestrians at a zebra crossing were included in the logistic regression model. Following this, only the significant variables were retained for the final analysis. The structure of the logistic regression model is shown in equations (10). The details of the model are shown in Table 4.

10 $$\begin{aligned}Yiel{d}_{cyclist}&=1.01-0.04\:{V}_{{cyclist}_{entering}}-0.15\:TrajectoryChang{e}_{cyclist}\\ &\quad+0.67\:SpeedReductio{n}_{cyclist}-0.23\:Interactio{n}_{Proximity}+0.07\:PedOnZebra\end{aligned}$$

**Table 4:** Logistic regression analysis results.

	Estimate	Std. Errors	t value	*p*-value	95% CI	Odd ratio
Intercept	1.01	0.25	4.004	< 0.001	1.68–4.52	2.76
$\:{{V}}_{{{c}{y}{c}{l}{i}{s}{t}}_{{e}{n}{t}{e}{r}{i}{n}{g}}}$	–0.04	0.02	–2.343	0.021	0.92–0.99	0.96
$\:{T}{r}{a}{j}{e}{c}{t}{o}{r}{y}{C}{h}{a}{n}{g}{{e}}_{{c}{y}{c}{l}{i}{s}{t}}$	–0.15	0.06	–2.425	0.017	0.76–0.97	0.86
$\:{S}{p}{e}{e}{d}{R}{e}{d}{u}{c}{t}{i}{o}{{n}}_{{c}{y}{c}{l}{i}{s}{t}}$	0.67	0.06	10.360	< 0.001	1.72–2.22	1.96
$\:{I}{n}{t}{e}{r}{a}{c}{t}{i}{o}{{n}}_{{P}{r}{o}{x}{i}{m}{i}{t}{y}}$	–0.23	0.07	–3.283	0.0014	0.69–0.91	0.79
$\:{P}{e}{d}{O}{n}{Z}{e}{b}{r}{a}$	0.07	0.03	2.047	0.043	1.00–1.14	1.07

The final logistic regression model retained several significant variables. Higher speeds of cyclists entering the zone were associated with a lower likelihood of yielding to pedestrians, indicating that faster cyclists are less inclined to yield. Cyclists who changed their path were also less likely to yield, suggesting that path changes correlate with non-yielding behavior. Conversely, cyclists who reduced their speed were significantly more likely to yield, implying that encouraging speed reduction could improve yielding behavior. Far interactions decreased the likelihood of yielding, indicating that cyclists are less likely to yield when pedestrians are not near the conflict point. Additionally, the presence of pedestrians already on the zebra crossing increased the likelihood of cyclists yielding, suggesting that visible pedestrian presence on the crossing prompts cyclists to yield.

All predictors exhibit very low variance-inflation factors (VIF ≈ 1.1–1.8), far below the commonly cited cautionary threshold of 2.5–5. This indicates that none of the variables is strongly linearly correlated with the others, so multicollinearity is negligible. Consequently, the standard errors and odds-ratio estimates in the logistic model should be stable and reliable. The model fits the data well. Residual deviance falls from 25.91 in the null model to 6.06 with the predictors included, so about 77% of the deviance is explained. A very small AIC of 10.5 further indicates a parsimonious yet informative fit, suggesting that the model provides a good overall description of the factors that influence whether cyclists yield at the crossing. The WebPower^39^ analysis indicates that a minimum of 55 observations is required to achieve the conventional 80% statistical power. With the actual sample of 107 cyclist-pedestrian interactions, the model exceeds this minimum threshold.

### Linear regression analysis

The linear regression analysis aimed to understand the relationship between cyclist obstructed travel time (OTT) and various predictors. Initially, all potential variables influencing cyclist OTT were included in the linear regression model. After identifying the significant predictors, the model was refined to retain only those variables. The structure of the linear regression model is shown in equations (11). The details of the model are shown in Table 5.

11 $$\begin{aligned}OTT&=4.20+0.92\:TTCP-0.44\:{V}_{{cyclist}_{entering}}-0.71\:De{c}_{cyclist}-0.49\:Di{r}_{cyclist}\\ & \quad +1.83\:SpeedReductio{n}_{cyclist}-2.02\:Interactio{n}_{Proximity}\end{aligned}$$

**Table 5:** Linear regression analysis results.

	Estimate	Std. errors	t value	*p*-value	95% CI
Intercept	4.20	1.10	3.816	< 0.001	2.02 to 6.38
$\:{T}{T}{C}{P}$	0.92	0.06	14.045	< 0.001	0.79 to 1.05
$\:{{V}}_{{{c}{y}{c}{l}{i}{s}{t}}_{{e}{n}{t}{e}{r}{i}{n}{g}}}$	–0.44	0.10	–4.426	< 0.001	–0.64 to –0.24
$\:{D}{e}{{c}}_{{c}{y}{c}{l}{i}{s}{t}}$	–0.71	0.26	–2.715	0.0078	–1.24 to –0.19
$\:{D}{i}{{r}}_{{c}{y}{c}{l}{i}{s}{t}}$	–0.49	0.23	–2.129	0.0357	–0.95 to –0.03
$\:{S}{p}{e}{e}{d}{R}{e}{d}{u}{c}{t}{i}{o}{{n}}_{{c}{y}{c}{l}{i}{s}{t}}$	1.83	0.38	4.756	< 0.001	1.07 to 2.60
$\:{I}{n}{t}{e}{r}{a}{c}{t}{i}{o}{{n}}_{{P}{r}{o}{x}{i}{m}{i}{t}{y}}$	–2.02	0.36	–5.622	< 0.001	–2.73 to –1.30

Residual standard error: 1.177 on 100 degrees of freedom.

Multiple R-squared: 0.8786, Adjusted R-squared: 0.8713.

F-statistic: 120.7 on 6 and 100 DF, p-value: < 2.2e-16.

The final linear regression model retained several significant variables. Pedestrian Time to Conflict Point (TTCP) is positively associated with OTT, indicating that as the time it takes for a pedestrian to reach the conflict point increases, the obstructed travel time for cyclists also increases. A larger OTT can suggest that cyclists are yielding to pedestrians, as they should by law. Higher speeds of cyclists entering the zone are associated with a decrease in OTT, suggesting that faster cyclists are less likely to yield and instead opt to pass quickly through the crossing, reducing their obstructed travel time. Greater deceleration by cyclists is associated with a reduction in OTT, indicating that cyclists who decelerate more abruptly tend to clear the crossing faster, potentially not yielding to pedestrians. The direction of travel of the cyclist is a significant predictor, with a negative estimate, suggesting that certain directions might influence cyclists’ likelihood of yielding. Cyclists who reduce their speed have significantly longer obstructed travel times, indicating that cyclists who slow down are yielding to pedestrians, resulting in increased OTT. The proximity of the interaction has a significant negative impact on OTT, with more far interactions leading to shorter obstructed travel times, possibly due to more decisive actions taken by cyclists to navigate through the conflict zone without yielding.

Pair-wise correlations among the three continuous predictors are modest (|r| ≤ 0.44), and all variance-inflation factors lie between 1.0 and 2.7, well below the usual cautionary threshold of 5. Hence multicollinearity is negligible, and each variable contributes largely unique information to the model. The regression model explains almost 88% of the variance in OTT (R^2^ = 0.879, adjusted R^2^ = 0.871) and the F-test is highly significant. Residuals have a standard error of 1.18 s and an AIC of 347, indicating a parsimonious yet powerful specification. With the observed effect size (f^2^ = 7.24) the current sample of 107 interactions yields virtually 100% statistical power. Even for a more conservative, medium effect (f^2^ = 0.15), the model would require 98 observations; the study exceeds this a-priori threshold, confirming that the sample is more than adequate for detecting meaningful relationships.

### Clustering

Model selection was considered for K = 1–5 under several covariance structures. Both BIC and ICL consistently selected K = 2 as the most parsimonious solution. Solutions with K ≥ 3 did not significantly improve the fit. These solutions tended to create a small third cluster composed primarily of observations close to the decision boundary in the TTCP-OTT space. This reduced interpretability without providing clear behavioral separation. Posterior assignment probabilities were generally high, indicating good separation for the two-group solution given the available data. For clarity and consistency in the remainder of this section, cyclists who yielded are referred to as “yielder” ($\:{Yield}_{cyclist}=Yes$), while those who did not yield are referred to as “non-yielders” ($\:{Yield}_{cyclist}=No$). Cluster 1 is referred to as non-yielders and Cluster 2 as yielders.

**Fig. 3:**
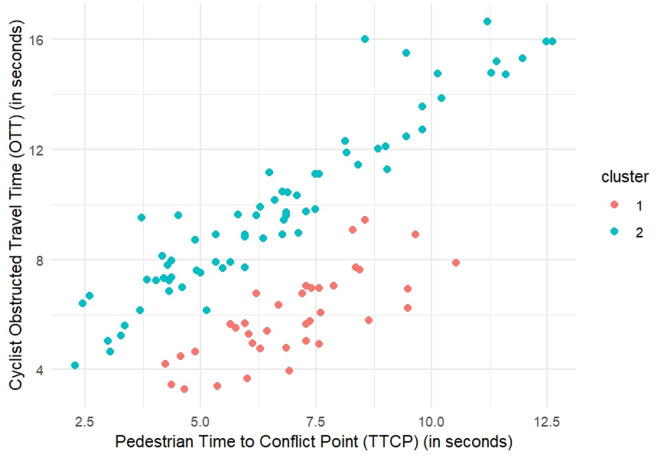
Clustering results for Mclust.

Using the Mclust method^37^, the Gaussian finite mixture model with ellipsoidal, equal orientation (VVE) was fitted, resulting in two optimal clusters. The model’s log-likelihood was − 456.4157, with a BIC of –959.5596 and an ICL of –964.0836. The clustering table shows that Cluster 1 contains 37 observations, while Cluster 2 contains 70 observations. The scatter plot generated from the Mclust clustering results displays two distinct clusters based on TTCP and OTT, as shown in Fig. 3. Cluster 1, non-yielder, (red in Fig. 3) primarily consists of data points with shorter TTCP and OTT values, indicating quicker interactions between cyclists and pedestrians. In contrast, Cluster 2, yielders, (blue in Fig. 3) consists of data points with longer TTCP and OTT values, suggesting more extended interactions where cyclists are more likely to yield to pedestrians. Tables 6 and 7 present the summary statistics for continuous and categorical variables, respectively, in each cluster.

**Table 6:** Summary of continuous variables per cluster.

Variables	Cluster 1			Cluster 2		
	Min	Max	Mean	Min	Max	Mean
$\:OTT\:\left(sec\right)$	3.28	9.44	5.89	4.16	16.64	9.85
$\:TTCP\:\left(sec\right)$	4.24	10.52	6.99	2.28	12.6	6.61
$\:{V}_{{cyclist}_{entering}}\:\left(m/s\right)$	2.12	9.01	5.67	2.62	7.62	4.96
$\:{Dec}_{cyclist}\left(m/{s}^{2}\right)$	–1.83	–0.02	–0.46	–2.85	–0.19	–1.22
$\:{V}_{ped}\left(m/s\right)$	0.89	3.30	1.8	0.42	3.30	1.56
Number of observations	37			70		

**Table 7:** Summary of categorical variables per cluster.

Variable		Cluster 1		Cluster 2	
	Category	Count	Percentage	Count	Percentage
$\:{Dir}_{cyclist}$	A	17	46%	32	46%
	B	20	54%	38	54%
$\:{Dir}_{ped}$	A	19	51%	27	39%
	B	18	49%	43	61%
$\:{Cross}_{inside}$	Yes	4	11%	60	86%
	No	33	89%	10	14%
$\:{Group}_{ped}$	Yes	9	24%	18	26%
	No	28	76%	52	74%
$\:PedOnZebra$	Yes	11	30%	52	74%
	No	11	30%	6	9%
	Ped started crossing	15	40%	12	17%
$\:{TrajectoryChange}_{cyclist}$	Yes	9	24%	14	20%
	No	28	76%	56	80%
$\:{SpeedReduction}_{cyclist}$	Yes	4	11%	63	90%
	No	33	89%	7	10%
$\:Interactio{n}_{Proximity}$	Close	13	35%	68	97%
	Far	24	65%	2	3%
$\:{Yield}_{cyclist}$	Yes	0	0%	7	10%
	No	37	100%	63	90%

**Fig. 4:**
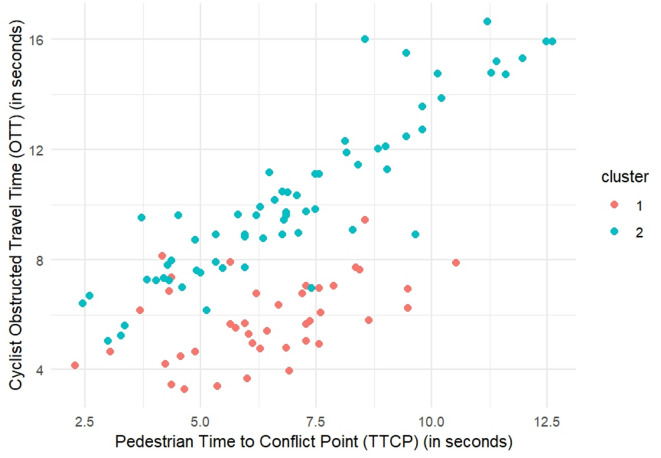
Clustering results for ClustMD.

The ClustMD method^33^ was also used to cluster the data, employing an EVI model with two components. The estimated BIC for this model is -2940.229. The clustering table for ClustMD indicates that Cluster 1 (non-yielders) contains 37 observations, while cluster 2 (yielders) contains 70 observations. Similar to the Mclust results, the scatter plot from the ClustMD clustering shows two distinct clusters based on TTCP and OTT as shown in Fig. 4. Cluster 1, non-yielders, (red in Fig. 4) includes data points with shorter TTCP and OTT values, suggesting faster interactions and less likelihood of yielding. In contrast, Cluster 2, yielders, (blue in Fig. 4) includes data points with longer TTCP and OTT values, indicating more time spent in the interaction, implying a more extended interaction, likely due to cyclists yielding more frequently to pedestrians.

**Fig. 5:**
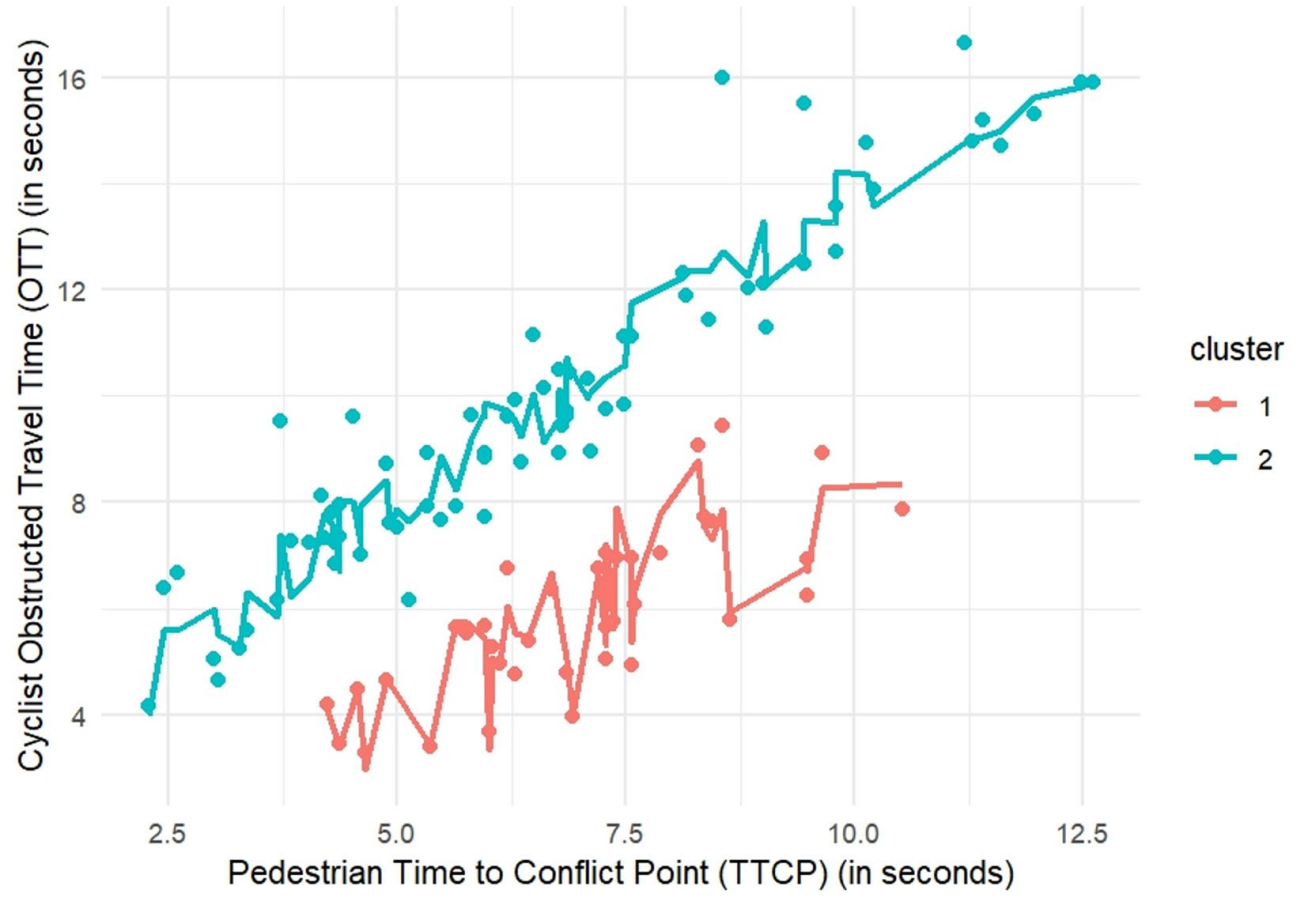
Results of mclust with separate regression models.

To deepen the analysis, the data was first clustered using the Mclust algorithm using only TTCP, which revealed two distinct groups. Within each cluster, a separate multiple linear regression model was fitted to explain the cyclist obstructed travel time (OTT) from TTCP and the additional predictors that were significant in the prior model summarized in equations (11). The predictions from these regression models are visualized in Fig. 5, illustrating how the relationships between predictors and OTT differ between the two clusters. The colored lines are the fitted values obtained from those cluster-specific regression models. Each line is drawn by ordering the observations in a cluster by TTCP and joining their model-predicted OTT, so it represents the trend predicted by the full multivariable model rather than a simple univariate smoothing.

The clustering table shows that cluster 1, non-yielders, consists of data points with shorter OTT, while cluster 2, yielders, include data points with longer OTT values. The regression model for Cluster 1, non-yielders, reveals that cyclists in this group generally have lower OTT values, suggesting they are less likely to yield or are obstructed by pedestrians for a shorter time. The variability in the regression line within Cluster 1, non-yielders, indicates that other factors, such as $\:{V}_{{cyclist}_{entering}}$, $\:De{c}_{cyclist}$, and $\:{Interaction}_{Proximity}$ also influence the OTT within this cluster. On the other hand, the regression model for Cluster 2, yielders, demonstrates a significant positive relationship between TTCP and OTT. This suggests that even when TTCP increases, OTT also increases. This indicates that cyclists in this cluster are more likely to wait and yield to pedestrians, resulting in longer OTT values.

## Discussion

The joint regression results highlight speed-related variables as the most significant predictor of cyclist behavior at the zebra crossing. Cyclists who reduced their speed in advance were more likely to yield, whereas higher entry speed, far cyclist-pedestrian interaction proximity, and any lateral swerve reduced the odds of yielding and shortened OTT. These findings align with previous research showing that lower speeds give road users more time to react to pedestrians, and thus lead to safer interactions^40^, and that cyclists often swerve to avoid yielding or colliding with pedestrians^41^.

Clustering methods offer a way to move beyond mean effects and uncover discrete behavioral groups in the data. Both Mclust and ClustMD independently converged on the same two behavioral groups, showing that unsupervised learning can reliably extract distinct profiles from trajectory data. Non-yielders (Cluster 1) cleared the crossing rapidly, exhibiting short OTT, high entry speed, and minimal deceleration, and suggesting that they are less likely to yield to pedestrians, possibly attempting to pass quickly before the pedestrians fully engage the crossing. Yielders (Cluster 2), on the other hand, included cyclists with longer OTT, greater speed reductions and deceleration, indicating deliberate yielding and greater respect for pedestrians’ right of way.

Although clustering yields useful behavioral groupings, the labels describe observed interaction patterns rather than fixed cyclist types. Posterior assignment probabilities were generally high, indicating good separation, but a small subset of observations lies near the decision boundary in the TTCP-OTT space. These borderline cases suggest locally continuous behavior between non-yielders and yielders and should be interpreted with caution.

Despite both clustering methods identifying two distinct clusters, the exact assignment of data points to clusters differs slightly between Mclust and ClustMD. Several factors could explain these differences. Mclust assumes data points are generated from a Gaussian mixture model and fits ellipsoidal clusters with equal orientation, capturing certain types of variability in the data. ClustMD incorporates continuous and categorical variables, using a mixture of Gaussian and discrete distributions. The EVI model allows for varying volume and equal shape, fitting data with different underlying distributions more effectively. Mclust treats all variables as continuous and applies Gaussian mixtures directly to the data, while ClustMD differentiates between continuous, ordinal, and categorical variables, leading to different clustering structures, especially if the data contains a mix of variable types. The initialization strategies and convergence criteria differ between the two methods, with Mclust using the EM algorithm for Gaussian mixtures, and ClustMD possibly using hierarchical clustering for initialization and a different approach to convergence, leading to different local optima. Differences in how the two methods handle scaling and normalization of variables can also affect the clustering outcome, with ClustMD applying different scaling strategies for continuous versus categorical data.

The Bayesian Information Criterion (BIC) indicated that two clusters best fit the data, neatly separating cyclists into yield and no-yield groups. However, this result may be influenced by the size of the dataset, the specific characteristics of the zebra crossing studied, or unobserved influences. For instance, pedestrian behavior such as assertiveness or distraction by mobile phones, and environmental conditions such as glare, rain on the pavement, or background vehicle noise may push a cyclist toward either cluster without being captured in the model. Because such confounding factors varied only narrowly in the current recordings, their influence cannot be separated from the core variables that drive the classification. With a larger dataset or data collected from other sites that exhibit wider ranges of pedestrian and cyclist behavior and environmental conditions, additional classifications might emerge, such as consistent yielders, opportunistic yielders, and non-yielders. This potential differentiation could offer a more detailed understanding of cyclist behavior and help guide the design of more targeted interventions.

Although yielding by cyclists likely exists along a continuum, the information-criterion analysis of this dataset supports two dominant behavioral modes: non-yielders, who approach faster with minimal deceleration, and yielders, who approach more slowly with a significant reduction in speed. Three-cluster exploratory solutions primarily separated a small borderline group near the boundary between these modes but did not improve model fit or interpretability. With larger, multi-site samples and broader variation in conditions, additional stable subgroups may emerge.

By revealing two distinct behavioral patterns, the clustering approach underscores the need for targeted interventions aimed at ensuring cyclist compliance with yielding laws at zebra crossings. The presence of a significant group of cyclists who do not yield poses safety risks to pedestrians. Interventions such as enhanced signage, speed reduction measures (e.g., bike-friendly speed bumps), improved intersection design, public awareness campaigns, and stricter enforcement of traffic laws could be effective in promoting safer behaviors. By focusing on the factors that influence whether cyclists yield to pedestrians, traffic management strategies can be more effectively designed to enhance pedestrian safety at unsignalized intersections.

Translating these findings into practice requires context-sensitive selection and piloting of measures rather than a one-size-fits-all approach. Enhanced signage is inexpensive and quick to deploy, but it is often less effective without physical speed management. Speed reduction measures for cyclists, such as bike-friendly speed bumps, can lower approach speeds. However, careful design is required to avoid discomfort or evasive swerving, and maintenance needs may constrain them. Improved intersection designs, such as narrowed approaches, better sight lines, and raised elements, tend to be more durable, but they require space, funding, and coordination with access needs. Public awareness campaigns are both scalable and visible. However, their impact typically fades without periodic repetition. Stricter enforcement can raise short-term compliance, but it requires sustained resources and consistent application. Overall, selecting and combining measures should reflect local site conditions and operational constraints. Despite the challenges, these measures can yield meaningful benefits, including lower approach speeds, higher yielding rates, improved pedestrian comfort, and clearer right-of-way expectations, especially when combined and periodically monitored.

Although the present dataset is limited to a single zebra crossing in Germany, the methodology is transferable to other urban settings and to different types of road user interactions. Drone footage or video for fixed pole-mounted cameras can be collected at signalized or unsignalized intersections, shared-space, and mid-block crossings. From these videos, key road user points can be extracted from trajectory data, local predictors can be identified, and behavior can be clustered like this study. Because the workflow is modular and data-driven, cities can generate comparable evidence even where crash records are sparse, enabling context-specific yet methodologically consistent safety interventions.

## Conclusions

This study established a drone-based, crash independent framework and proof of concept for analyzing cyclist-pedestrian interactions at an unsignalized zebra crossing. By using high-resolution footage captured from more than 60 m above the carriageway, the method eliminates the occlusion, observer-effect, and privacy hurdles that hinder camera video data, while still preserving naturalistic behavior and high positional accuracy. This approach is particularly valuable for locations where crash data is sparse, underreported, or difficult to obtain, such as unsignalized intersections. The surrogate safety measures, regression models, and behavior-based clusters presented here provide an alternative method for assessing safety at intersections, enabling proactive safety measures in areas where crash data is unavailable. Although pedestrian fatalities involving cyclists are rare, the discomfort and hesitation many pedestrians experience when cyclists fail to yield underline the need for such forward-looking approaches.

Only 59% of cyclists yielded to pedestrians at the zebra crossing, 29% of them did so before pedestrians had started crossing, while 41% of cyclists did not yield at all, highlighting a significant level of non-compliance. Of those who failed to yield, 45% had close interactions with pedestrians, and 75% did not yield when pedestrians were already on the zebra crossing. Regression analysis showed that speed-related variables are the primary predictors of cyclist-pedestrian interactions. Logistic regression showed that cyclists traveling at higher speeds or those who changed their trajectory were less likely to yield, whereas speed reduction and pedestrian presence on the crossing increased the likelihood of yielding. Linear regression further showed this pattern: faster cyclists and abrupt deceleration were associated with shorter OTT, indicating less yielding, while speed reduction was positively associated with longer OTT, suggesting that encouraging cyclists to reduce speed could promote yielding behavior. Clustering analysis using Mclust and ClustMD revealed two optimal and distinct groups of cyclist behavior. Non-yielders (Cluster 1) exhibited shorter OTT, reflecting lower levels of yielding, while yielders (Cluster 2) had longer OTT, indicating a higher likelihood of yielding. This framework situates TTCP and OTT within the broader family of surrogate safety measures, highlighting how SSMs enable proactive safety assessment when crash data is limited. Hence, the findings should be interpreted as proactive screening evidence to guide design and operational choices rather than as direct estimates of crash risk.

This differentiation suggests that targeted interventions are needed to ensure compliance with yielding laws, particularly for non-yielders (Cluster 1), who are less likely to yield. By focusing on the significant predictors identified, traffic management strategies can be more effectively designed to protect pedestrians and enhance safety at unsignalized intersections. Interventions encouraging cyclists to slow down – such as “Yield to Pedestrian” markings or gentle speed cushions – and ensuring pedestrian visibility could enhance safety at zebra crossings. Clearer crossing designs may also help cyclists anticipate pedestrian presence and react appropriately. Educating cyclists about the importance of yielding and the potential consequences of not doing so could also be beneficial, especially targeting non-yielders (Cluster 1).

While this study provides valuable insights into cyclist-pedestrian interactions at an unsignalized intersection, several limitations should be acknowledged. First, the focus on a single German intersection, influenced by the availability of high-quality data, limits the transferability of the findings to other intersections with different environmental or design characteristics. In particular, the studied site is an unsignalized zebra crossing on a two-way street with one lane in each direction. The design and operational features of the intersection, such as the approach geometry and sight distance, the crosswalk length and marking style, as well as pedestrian and cyclist volumes, can influence cyclist yielding behavior and perceived risk. Moreover, local cultural context and enforcement norms may influence yielding expectations. While cyclists may perceive non-yielding as low risk when acceptable gaps exist, pedestrians may still experience discomfort, hesitation, or reduced perceived safety. The findings should be interpreted with caution when applied to settings that are different from those in which they were observed. Second, although an a-priori power check indicated that 107 interactions are sufficient to detect medium effect sizes in the regression models, this is still a modest sample. A larger sample size is therefore essential for testing the stability of the regression models and cluster boundaries. Third, the dataset.

lacks demographic information such as age, gender and socioeconomic status. Because different user groups exhibit systematic differences, the absence of these variables can bias effect estimates and further limit generalizability. Fourth, key contextual factors, such as sun position/glare, surface condition, ambient noise, and distraction indicators (e.g., phone use), were unavailable. These unmeasured variables may confound observed associations and influence cluster assignment. Due to the privacy constraints inherent to top-view video, direct measurement of these attributes is limited, which limits interpretability. Fifth, although the trajectories supplied by the source dataset were refined with Bayesian smoothing and claim pixel-level positional accuracy, residual noise and the interpolation of occluded frames can still shift the key timestamps ($\:{P}_{1}$, $\:{P}_{2}$, and $\:{P}_{3}$) by a few tenths of a second, injecting random error into OTT and TTCP. Finally, this study relies on trajectory-derived surrogate safety measures (TTCP and OTT). Although SSMs allow for a proactive assessment in areas with limited crash data, they are proxies rather than direct measures of crash risk. Their values depend on the zone definitions, timestamp estimations, and site context. Accordingly, the results should be interpreted as indicators of interaction dynamics and compliance rather than as calibrated crash predictions. Future work should examine transportability across sites and, where feasible, validate the results against independent conflict logs or multi-site datasets.

Future research should expand the scope to include data from multiple intersections with varying widths and flow levels, larger sample sizes, and demographic information to provide a broader understanding of cyclist-pedestrian interactions. To improve generalizability, future research should explicitly sample intersections with varied lane configurations, including multi-lane approaches, as well as different facility types and cultural contexts. Additionally, it should pair trajectory-based outcomes with pedestrian-reported measures of comfort and perceived safety. To address measurement gaps under privacy constraints, future work should explore alternative video modalities and viewpoints. For example, infrared or thermal imagery, as well as oblique or multi-camera angles, could be employed to detect the use of mobility aids and group composition without identifying faces. Additionally, future work should use time of day, solar position, and local weather data as proxies for glare and surface conditions. Additionally, combining trajectories with open environmental datasets (e.g., land use, facility type) would provide a better context for behavior. Additionally, the influence of vehicles and the impact of different infrastructure designs, such as bike lanes, mid-block crossings, and pedestrian islands, should be explored to assess their effectiveness in improving safety at unsignalized intersections. Examining cyclist behavior during right and left turns, as well as the role of advanced technologies like intelligent traffic signals and real-time warning systems, could offer new ways to manage cyclist behavior and enhance safety. Furthermore, it is important to consider the dynamics of following cyclists, not just leading ones, to fully capture cyclist-pedestrian interactions at zebra crossings.

## Data Availability

The trajectory and video data analyzed in this study are drawn from the inD dataset. They can be accessed by contacting the LevelXData team using the “Application for Access for Non-Commercial Use” form found at the bottom of the dataset webpage (https://levelxdata.com/ind-dataset/). Once the form is submitted, it is automatically forwarded to the LevelXData team, who will email the requester a download link after the request is approved. For assistance with reproducing the data transformations used in this study, please contact the corresponding author (hiba.nassereddine@unibw.de).

## References

[1] Garrard, J., Rissel, C. & Bauman, A. & Giles-Corti, B. Cycling and health. (2021).

[2] Pucher, J., Parkin, J. & de Lanversin, E. Cycling in New York, London, and Paris. (2021).

[3] Blitz, A., Busch-Geertsema, A. & Lanzendorf, M. More Cycling Less Driving? Findings of a Cycle Street Intervention Study in the Rhine-Main Metropolitan Region, Germany. *Sustainability* **12**, 805 (2020).

[4] Lanzendorf, M. & Busch-Geertsema, A. The cycling boom in large German cities—empirical evidence for successful cycling campaigns. *Transp. Policy*. **36**, 26–33 (2014).

[5] Abduljabbar, R. L., Liyanage, S. & Dia, H. The role of micro-mobility in shaping sustainable cities: a systematic literature review. *Transp. Res. Part. D: Transp. Environ.* **92**, 102734 (2021).

[6] Cloutier, M. S. et al. Outta my way! Individual and environmental correlates of interactions between pedestrians and vehicles during street crossings. *Accid. Anal. Prev.* **104**, 36–45 (2017). 28482177 10.1016/j.aap.2017.04.015

[7] Paudel, M. et al. A computational study on the basis for a safe speed limit for bicycles on shared paths considering the severity of pedestrian head injuries in bicyclist-pedestrian collisions. *Accid. Anal. Prev.* **176**, 106792 (2022). 35952395 10.1016/j.aap.2022.106792

[8] Muggenthaler, H., Drobnik, S., Hubig, M., Fiebig, W. & Mall, G. Fatal abdominal injuries in a bicycle-pedestrian collision—reconstruction using multibody simulation. *Forensic Sci. Med. Pathol.* **13**, 230–233 (2017). 28409387 10.1007/s12024-017-9866-5

[9] Tuckel, P., Milczarski, W. & Maisel, R. Pedestrian injuries due to collisions with bicycles in New York and California. *J. Saf. Res.* **51**, 7–13 (2014). 10.1016/j.jsr.2014.07.003 25453171

[10] Haworth, N., Schramm, A. & Debnath, A. K. An observational study of conflicts between cyclists and pedestrians in the city centre. Journal of the Australasian College of Road Safety 25(4), 31–40 (2014)

[11] Beitel, D., Stipancic, J., Manaugh, K. & Miranda-Moreno, L. Assessing safety of shared space using cyclist-pedestrian interactions and automated video conflict analysis. *Transp. Res. Part. D: Transp. Environ.* **65**, 710–724 (2018).

[12] Zhang, C., Du, B., Zheng, Z. & Shen, J. Space sharing between pedestrians and micro-mobility vehicles: a systematic review. *Transp. Res. Part. D: Transp. Environ.* **116**, 103629 (2023).

[13] Luo, L., Luo, Y., Feng, Y., Li, T. & Fu, Z. Experimental investigation on pedestrian–bicycle mixed merging flow in T-junction. *Phys. A: Stat. Mech. Its Appl.* **600**, 127492 (2022).

[14] Li, B., Xiong, S., Li, X., Liu, M. & Zhang, X. The behavior analysis of Pedestrian-cyclist interaction at non-signalized intersection on campus: conflict and interference. *Procedia Manuf.* **3**, 3345–3352 (2015).

[15] Hatfield, J. & Prabhakharan, P. An investigation of behaviour and attitudes relevant to the user safety of pedestrian/cyclist shared paths. *Transp. Res. Part. F: Traffic Psychol. Behav.* **40**, 35–47 (2016).

[16] Bundesministerium für Digitales und Verkehr (BMDV). Cycling Monitor. (2023). https://bmdv.bund.de/SharedDocs/EN/Articles/StV/Cycling/cycling-monitor.html

[17] Hosford, K., Cloutier, M. S. & Winters, M. Observational study of pedestrian and cyclist interactions at intersections in Vancouver, BC and Montréal, QC. *Transp. Res. Rec.* **2674**, 410–419 (2020).

[18] Mesimäki, J. & Luoma, J. Near accidents and collisions between pedestrians and cyclists. *Eur. Transp. Res. Rev.* **13**, 38 (2021).

[19] Abdulhafedh, A. Road traffic crash data: an overview on sources, problems, and collection methods. *JTTs*. **07**, 206–219 (2017).

[20] Vedagiri, P. & Kadali, B. R. Evaluation of pedestrian–vehicle conflict severity at unprotected midblock crosswalks in India. *Transp. Res. Rec.* **2581**, 48–56 (2016).

[21] Gettman, D. & Head, L. Surrogate safety measures from traffic simulation models. *Transp. Res. Rec.* **1840**, 104–115 (2003).

[22] Saunier, N. & Sayed, T. A feature-based tracking algorithm for vehicles in intersections. in *The 3rd Canadian Conference on Computer and Robot Vision (CRV’06)* 59–59IEEE, Quebec, Canada, (2006). 10.1109/CRV.2006.3

[23] Nassereddine, H., Santiago-Chaparro, K. R. & Noyce, D. A. Modeling vehicle–pedestrian interactions using a nonprobabilistic regression approach. *Transp. Res. Rec.* **2675**, 356–364 (2021).

[24] Nassereddine, H., Santiago-Chaparro, K. R. & Noyce, D. A. Evaluating right-turn flashing yellow arrow for vehicle–pedestrian interactions using a non-probabilistic regression approach. *Transp. Res. Record: J. Transp. Res. Board.* **2678**, 212–222 (2024).

[25] Nassereddine, H. Modeling vehicle-pedestrian interactions at unsignalized intersections. *J. Transp. Saf. Secur.* **1–19**10.1080/19439962.2024.2447989 (2025).

[26] Kathuria, A. & Vedagiri, P. Evaluating pedestrian vehicle interaction dynamics at un-signalized intersections: a proactive approach for safety analysis. *Accid. Anal. Prev.* **134**, 105316 (2020). 31677475 10.1016/j.aap.2019.105316

[27] Danielsson, S., Gustafsson, S., Hageback, C., Johansson, U. & Olsson, C. Korsningen Radhusgatan-Storgatan, seminarieuppgift i Trafikanalys. *Tekniska Hogskolan I Lulea Sweden* (1993).

[28] Hydén, C., Odelid, K. & Varhelyi, A. Effekten av generell hastighetsdämpning i tätort. Resultat av ett storskaligt försök i Växjö. I. Huvudrapport. *3109/3000* (1995).

[29] Lehká, E. Bezpečnost chodc\uu Na přechodech pro Chodce v České Republice a Dánsku. (České vysoké učení technické v praze. (2015). Vypočetní a informační centrum.

[30] Sogbe, E. An investigation into drivers’ yielding behaviour at marked uncontrolled pedestrian crossings in Ghana. *IATSS Res.* **48**, 100–107 (2024).

[31] Sucha, M., Dostal, D. & Risser, R. Pedestrian-driver communication and decision strategies at marked crossings. *Accid. Anal. Prev.* **102**, 41–50 (2017). 28259827 10.1016/j.aap.2017.02.018

[32] Gormley, I. C., Murphy, T. B. & Raftery, A. E. Model-based clustering. *Annual Rev. Stat. Its Application*. **10**, 573–595 (2023).

[33] McParland, D. & Gormley, I. C. Model based clustering for mixed data: ClustMD. *Adv. Data Anal. Classif.* **10**, 155–169 (2016).

[34] Kutner, M. H., Nachtsheim, C. J., Neter, J. & Li, W. *Applied Linear Statistical Models* (McGraw-hill, 2005).

[35] Hosmer, D. W. Jr, Lemeshow, S. & Sturdivant, R. X. *Applied Logistic Regression* (Wiley, 2013).

[36] Wang, T., Qin, L., Dai, C., Wang, Z. & Gong, C. Heterogeneous learning of functional clustering regression and application to Chinese air pollution data. *IJERPH* **20**, 4155 (2023). 36901175 10.3390/ijerph20054155 PMC10002127

[37] Scrucca, L., Fraley, C., Murphy, T. B. & Raftery, A. E. *Model-Based Clustering, Classification, and Density Estimation Using Mclust in R* (Chapman and Hall/CRC, 2023).

[38] Bock, J. et al. The ind dataset: A drone dataset of naturalistic road user trajectories at german intersections. in 2020 IEEE Intelligent Vehicles Symposium (IV) 1929–1934 (IEEE, 2020)

[39] Zhang, Z., Mai, Y., Yang, M. & WebPower Basic and advanced statistical power analysis. *R package version 0.5* 2, (2018).

[40] Schroeder, B. J. & Rouphail, N. M. Event-Based modeling of driver yielding behavior at unsignalized crosswalks. *J. Transp. Eng.* **137**, 455–465 (2011). 21852892 10.1061/(ASCE)TE.1943-5436.0000225 PMC3156582

[41] Miyadai, M., Uetake, T. & Shimoda, M. How does a cyclist avoid obstacles? *J. Hum. Ergol.* **41**, 95–100 (2012). 25665202

